# Effects of Harvest Time on Grinding Quality, Appearance Quality and Physical and Chemical Quality of Japonica Rice

**DOI:** 10.3390/foods13182868

**Published:** 2024-09-10

**Authors:** Mengnan Teng, Xiaoliang Duan, Ru Feng, Dong Zhang, Weiqun Guo, Hui Sun, Xingquan Liu

**Affiliations:** 1 School of Food and Health Sciences, Zhejiang A&F University, Hangzhou 311302, China; tmn15968339133@163.com (M.T.); liuxq@zafu.edu.cn (X.L.); 2 Academy of National Food and Strategic Reserves Administration, Beijing 100037, China; dxl@ags.ac.cn (X.D.); gwq@ags.ac.cn (W.G.); sh@ags.ac.cn (H.S.); 3 Suzhou Suken Modern Agricultural Development Co., Ltd., Suzhou 215331, China; nkscbfr@live.cn

**Keywords:** japonica rice (*Oryza sativa* L.), harvest time, quality, accumulated temperature, growing region

## Abstract

Harvest time is very important to rice due to its high correlation to rice yield, eating quality, etc.; however, the impact of harvest time on quality is still unclear. In this study, Nangeng 5718, a japonica rice planted in three regions in Jiangsu Province of China, was used to analyze and compare the milling quality, appearance quality, and physicochemical quality of japonica rice at different harvest times. The results showed that the 1000-grain weight of Nangeng 5718 exhibited no significant change at different harvest times (*p* > 0.05). The brown rice rate and rice yield at different harvest times were 82.3–85.4% and 66.3–76.1%, respectively. Harvest time had no significant effect on the brown of rice (*p* > 0.05). However, Nangeng 5718 planted in Nanjing had the highest rice yield at 50 days after heading, which was significantly different from that of rice harvested 65 days after heading (*p* < 0.05). Nangeng 5718 planted in Huai’an had the highest rice yield at 55 days after heading, which was significantly different from that of rice harvested 60 days after heading (*p* < 0.05). Harvest time had little effect on the length, width, and thickness of rice. The immature grain rate showed a decreasing trend with the increase in maturity. There were little differences in the protein content of Nangeng 5718 at different harvest times. Nangeng 5718 planted in Nanjing had the highest protein content at 50 days after heading. There was a significant difference between the rice harvested and the rice harvested 60 days after heading (*p* < 0.05). There were no significant differences between the other two regions (*p* > 0.05). The accumulated temperature in Nanjing was relatively high, and the RVA curve and RVA eigenvalues of rice varied greatly. The setback value of rice harvested at 50 days was significantly lower than that at 55 days and 60 days (*p* < 0.05). Rice has good gelatinizing properties. Therefore, timely harvesting and appropriate accumulated temperature could increase 1000-grain weight and rice yield, reduce the immature grain rate, and improve the gelatinization characteristics. Overall, the quality of Nangeng 5718 reached a good level when it was harvested 50 days after heading, with the accumulated temperature reaching 1051 °C. In fact, the harvest time should be chosen flexibly according to the weather conditions. Nangeng 5718 planted in Nanjing should be harvested earlier than 50 days, and rice from Huai’an and Lianyungang was of better quality when the harvest time was 50 days.

## 1. Introduction

In recent years, with the improvement of rice breeding and cultivation techniques, rice production has increased steadily [1]. However, in agricultural practice, either early or late harvesting could cause losses to the final rice yield and its eating quality. Generally, rice is usually harvested when 90–95% of the grains turn yellow after heading [2]. Li et al. [3] found that with the delay of harvest time, the 1000-grain weight of rice first increased and then decreased, and the 1000-grain weight reached the maximum when the grain was mature on the 55th day after heading. Rice quality is a very complex concept, which mainly includes appearance quality, processing quality, physical and chemical quality, and cooking quality [4]. Wang et al. [5] found that the rice grain filling was insufficient and there were relatively more green and immature grains when early harvest. Lu et al. [6] found that with the delay of harvesting time, the gelatinization property of Nangeng 3908 was improved, and the eating quality was better. Protein content is an important index to evaluate the physical and chemical quality of rice. Jiang et al. [7] found that the protein content was negatively correlated with the taste quality of rice, and the lower the protein content, the better the taste.

In agricultural practice, there are differences in the temperature changes of each planting site, so it is necessary to find the accumulated temperature required for the best maturity period in the local area and determine the best harvest time of rice. Lv et al. [8] demonstrated that there was a significant positive correlation between the taste quality of rice and the accumulated temperature from heading to harvest, which could appropriately delay the harvest date and improve the taste.

With the improvement of people’s living standards, people’s requirements for rice quality are becoming higher and higher. In order to improve the quality of rice on the premise of ensuring its yield, it is particularly important to produce high-quality rice. So far, there are few studies to find the optimal ripening time and optimal ripening temperature for japonica rice by changing the harvest time of rice to understand the relationship between accumulated temperature and rice quality from heading to harvesting. Nangeng 5718 is a new japonica rice variety of the Nangeng series cultivated by the Jiangsu Academy of Agricultural Sciences in China. Nangeng 5718 has the characteristics of vigorous growth, high spike formation rate, and lodging resistance [9]. Nanjing, Huai’an, and Lianyungang are all located in Jiangsu Province of China. The climatic conditions in the three regions are suitable for the growth of Nangeng 5718. The latitude of Nanjing, Huai’an, and Lianyungang gradually increased. There are differences in temperature between the three regions, which may affect the final quality.

In this study, Nangeng 5718 was selected as the research object, and the effects of different harvesting times on 1000-grain weight, brown rice rate, rice yield, immature grain rate, length, width and thickness, protein content, gelatinization characteristics, and other related indexes of rice from the cities of Nanjing, Huai’an, and Lianyungang of Jiangsu Province in China were analyzed and compared. Through the effect of harvest time on rice quality index, to provide a theoretical basis for determining the optimal harvest time of japonica rice.

## 2. Materials and Methods

### 2.1. Materials

**Figure 1:**
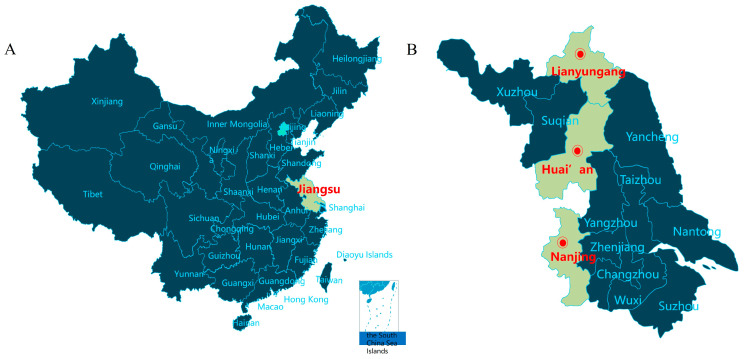
Rice cultivation sites. (**A**) Map of China, (**B**) Map of Jiangsu Province.

Rice samples (cv. Nangeng 5718) used in this study were provided by the Rice Research Institute of the Jiangsu Academy of Agricultural Sciences in China, which were selected from the cities of Nanjing, Huai’an, and Lianyungang of Jiangsu Province. Rice were sprouted in the plastic tray and then transplanted manually in the field with 3–4 seedlings per hole and shallow irrigation. Paddy rice was harvested at 50, 55, and 60 days after full heading. A total of 5 kg of rice was harvested at a 5 day interval. Rice samples were manually threshed and dried naturally. The rice cultivation sites are shown in Figure 1.

As shown in Table 1, the sowing time of Nangeng 5718 planted in Nanjing was 19 May 2023, the heading time was 19 August 2023, the accumulated temperatures of the three harvest times ranged from 1219 °C to 1409 °C, and the moisture content after drying was 9.05–10.29%. Nangeng 5718 planted in Huai’an was sown on 30 May 2023, and the heading time was 22 August 2023, with an accumulated temperature of 1143–1324 °C and a moisture content of 10.36–11.05% after drying. Nangeng 5718 planted in Lianyungang was sown on 22 May 2023, and the heading time was 3 September 2023, the accumulated temperature of the three harvest times was 1051–1219 °C, and the moisture content was 9.16–9.85% after drying.

### 2.2. Brown Rice Rate and Rice Yield

A total of 1 kg of paddy rice was weighed and hulled by a JDMZ100 hulling machine (Beijing Dongfu Jiuheng Instrument Technology Co., Ltd., Beijing, China) to obtain brown rice, which was then milled into white rice by a CBS300B rice mill (Satake, Japan) and passed through a 1.2 mm round hole sieve and weighed. Finally, the mass fraction of polished rice in brown rice samples was calculated [10,11]. Rice yield = (rice weight/brown rice weight) × 100%

### 2.3. Length-Width Ratio and Thickness

Ten brown rice grains were randomly selected and placed flat on the table. Brown rice was closely arranged in a line with head-to-head and tail-to-tail. The total length divided by 10 was the average length of the rice. Ten brown rice grains were randomly selected and lined back to back. The maximum total value of rice grains was measured with a DL 91150 vernier caliper (Deli Group Co., Ltd., Beijing, China). The total width divided by 10 was the average width of the rice. Twenty brown rice grains were randomly selected, and the thickest part was measured by an APU thickness gauge (Aipu Measuring Instrument Co., Ltd., Quzhou, China) [12,13].

### 2.4. Immature Grain Rate

A total of 20.0 g of brown rice were weighed with an ML 104-02 analytical balance (Shanghai Mettler Toledo Instrument Co., Ltd., Shanghai, China). Immature rice grains were selected and weighed with an ML 104-02 analytical balance (Shanghai Mettler Toledo Instrument Co., Ltd., Shanghai, China) to calculate the mass fraction of immature grains.

### 2.5. 1000-Grain Weight, Moisture, and Protein Content

An automatic grain counting instrument Numigral I (Beijing Michael Technology Co., Ltd., Beijing, China) was used to count 1000 grains of rice. Rice was weighed with an analytical balance to complete the determination of the 1000-grain weight of rice [14]. For moisture determination, rice flour was dried at 130 °C to a constant weight, and the quality lost after drying of brown rice flour was determined [15]. The protein content was determined by a DN2100 Dumas nitrogen analyzer (Beijing NordTech Instrument Co., Ltd., Beijing, China). A total of 100 mg of brown rice flour was weighed, wrapped in tin foil, and placed in the sample tray. The samples were delivered to the combustion reactor and burned at a high temperature of 900–1200 °C. The nitrogen oxides generated by combustion were reduced to nitrogen in the reduction furnace to obtain the protein content of the sample. The protein coefficient was 5.95 [16].

### 2.6. Gelatinization Properties

The gelatinization properties of rice were determined by an RVA-Super4 Rapid viscosity analyzer (Perten, Sageltorp, Sweden). A total of 25 mL of deionized water and 3 g of brown rice flour were added to the sample cylinder (corrected according to a 12% wet basis), and the stirrer was used to quickly stir up and down in the sample barrel for 10 times until the sample was completely dispersed, and the sample barrel was connected to the stirrer to drive the test program and complete the determination of the gelatinization characteristics of brown rice flour [17].

### 2.7. Data Statistics and Analysis

The data from the experiment were analyzed and mapped using SPSS 24.0 (IBM Corporation, Chicago, IL, USA) and origin 9 (OriginLab Corporation, Northampton, MA, USA) softwares. The data were statistically analyzed at the *p* < 0.05 test level, and the results were expressed as mean ± standard deviations.

## 3. Results and Discussion

### 3.1. Effect of Harvest Time on the Grinding Quality of Japonica Rice

#### 3.1.1. Brown Rice Rate

**Figure 2:**
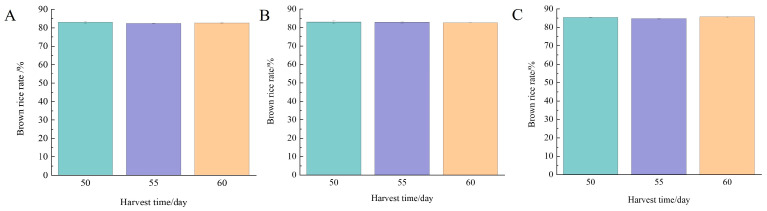
Brown rice rate at different harvest times. (**A**) Nanjing, (**B**) Huai’an, (**C**) Lianyungang.

According to Figure 2, there was no significant difference in the brown rice rate of Nangeng 5718 planted in Nanjing, Huai’an, and Lianyungang at different harvest time (*p* > 0.05). In addition, the brown rice rate of the three regions was 82.3–85.4%, all of which were above 81% and met the first-level requirements of China’s national standard [18]. Liu et al. [19] found that the brown rice rate of Nangeng 5055 and Nangeng 9108 planted in China showed an increasing trend with the postponement of harvest time and then decreased slightly after the peak growth. Liu et al. [20] found that with the postponement of harvesting, the brown rice rate of Jigeng 809 and Jigeng 88 gradually increased.

#### 3.1.2. Rice Yield

**Figure 3:**
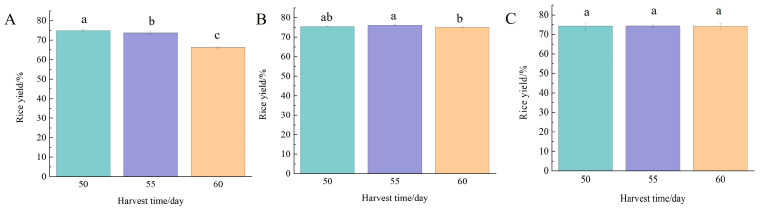
Rice yield at different harvest times. (**A**) Nanjing, (**B**) Huai’an, (**C**) Lianyungang. Different lowercase letters indicate inter-treatment differences are significant at the 0.05 level.

Rice yield rate refers to the mass fraction of rice as net rice after milling. It can be seen from Figure 3 that Nangeng 5718 planted in Nanjing was harvested at 50, 55, and 60 days after heading, and the rice yield showed a decreasing trend, while the rice yield of 50 days was significantly higher than that of the last two harvesting times (*p* < 0.05). Nangeng 5718 planted in Huai’an had the highest rice yield at 55 days after heading, which was significantly different from that of rice harvested 60 days after heading (*p* < 0.05). There was no significant difference in the rice yield of Nangeng 5718 planted in Lianyungang at three harvest times (*p* > 0.05). Therefore, Nangeng 5718 was harvested 50 and 55 days after heading, and the rice yield was higher. In addition, the rice yield of the three regions was 66.3–76.1%, all of which were above 61%, the first-level requirements of China’s national standard.

Gong et al. [21] showed that the harvest was too early, the grain fullness was low, the hardness was not enough, and the rice grains were easy to break during processing. If the harvest time was too late, the mature grains were repeatedly dried by absorbing water, leading to the loose starch arrangement, low hardness, and easy to fracture when processing. Consistent with the change in rice yield planted in Nanjing, the immature grain rate was zero when harvested 60 days after heading in this area, and the grain maturity was high and the harvest time was too late, so the rice yield was lower at 66.3%. Dou et al. [22] proved that high temperature increased the amount of broken rice, resulting in poor milled quality of rice. Zhou et al. [23] stated that temperature and solar radiation had a great impact on the milling quality of rice, but the temperature requirements of different rice varieties were different. The accumulated temperature in this area was high at 1219–1409 °C, and the milling quality of brown rice decreased. Zou et al. [24] found the yield of rice reached the highest when the grain was fully mature, and the yield decreased in early or late harvest. It was consistent with the change trend of rice yield in Huai’an.

### 3.2. Effect of Harvest Time on the Appearance of Japonica Rice

#### 3.2.1. Length, Width, and Thickness

Grain shape is an important indicator of appearance quality, which usually refers to the length, width, length-to-width ratio, and grain thickness of rice grains. It can be seen from Table 2 that different harvest times had little effect on the grain length, width, and length–width ratio of Nangeng 5718. However, the grain length and length–width ratio of Nangeng 5718 planted in Lianyungang were significantly lower than those of rice planted in Nanjing and Huai’an, and the accumulated temperature in the area was also significantly lower than that of the other two planting sites (1051–1219 °C), indicating that the accumulated temperature had an effect on the grain shape of brown rice. The results reported by Du et al. [25] showed that the delayed harvest had little effect on the grain shape of the three rice varieties of Nangeng 2728, Nangeng 9108, and Nangeng 3908 planted in China. Li et al. [26] also found that the length-width ratios of Yanfeng 47, Yanjing 218, Yanjing 456, H50, and H199 planted in China remained basically unchanged at each harvest period. It was consistent with the results of the aspect ratio of Nangeng 5718 in the three regions in this study.

The thickness of the rice grain indicates its fullness. There was no significant difference in the thickness of Nangeng 5718 planted in Nanjing (*p* > 0.05). The grain thickness of Nangeng 5718 planted in Huai’an and Lianyungang reached the maximum harvest time of 60 days after heading, and there was a significant difference from the previous two harvest times (*p* < 0.05). In conclusion, the grain thickness of brown rice of Nangeng 5718 showed an increasing trend with the increase in maturity. Matsue [27] believed that the grain thickness of brown rice was mostly in the range of 1.6–2.2 mm, and the taste quality tended to improve with the increase in grain thickness, but the taste change was small when the grain thickness was greater than 2.0 mm. In this study, the grain thickness of Nangeng 5718 planted in three regions was in the range of 2.24–2.33 mm, and the grain thickness reached a good level.

#### 3.2.2. Immature Grain Rate

**Figure 4:**
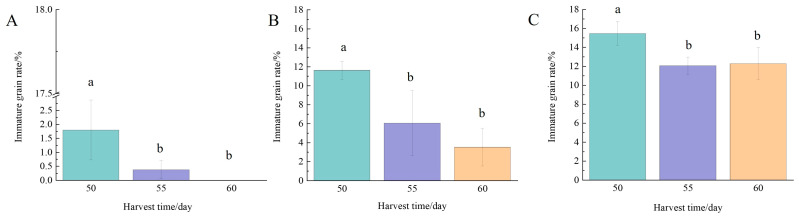
Immature grain rate at different harvest times. (**A**) Nanjing, (**B**) Huai’an, (**C**) Lianyungang. Different lowercase letters indicate inter-treatment differences are significant at the 0.05 level.

Immature grain rate are immature and not full grains, and the appearance of rice grains is all silty grains, which is usually the incomplete storage of nutrients in the rice itself. As can be seen from Figure 4, harvest time had a significant effect on the immature grain rate. The immature grain rate of Nangeng 5718 rice planted in the three regions was the highest at 50 days after heading, and the harvest time of the next two gradually decreased. Among them, the immature grain rate of Nangeng 5718 planted in Nanjing did not exceed 1.8%, and the accumulated temperature of the three harvest times in this area was relatively high at 1219–1409 °C, the rice grain was fully developed, and the maturity of rice was generally high. Li et al. [28] found that the immature grain rates of Tsuhara 45, Tsuhara E28, and Tsukawa 1 decreased significantly with the delay of harvesting time. The variation trend in Immature grain rate of Nangeng 5718 at different harvest times was consistent with that in this study.

### 3.3. Effect of Harvest Time on the Physical and Chemical Properties of Japonica Rice

#### 3.3.1. 1000-Grain Weight

**Figure 5:**
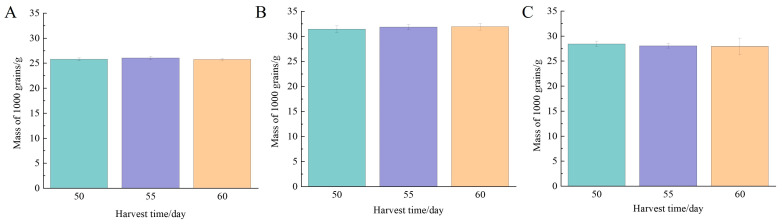
The 1000-grain weight at different harvest times. (**A**) Nanjing, (**B**) Huai’an, (**C**) Lianyungang.

The 1000-grain weight reflects the grain size and fullness of the rice. According to Figure 5, there was no significant difference in 1000-grain weight of Nangeng 5718 planted in Nanjing, Huai’an, and Lianyungang after heading (*p* > 0.05), indicating that the 1000-grain weight of rice reached a higher value when the accumulated temperature was 1051 °C. Wang et al. [29] showed that harvest time had a significant effect on dry matter accumulation of rice. Yong et al. [30] proved that with the postponement of harvest time, the 1000-grain weight of rice increased greatly in the early stage and then gradually stabilized and decreased slightly. Lin et al. [31] also found that with the postponement of harvesting, the 1000-grain weight gradually increased, and the 1000-grain weight reached the maximum value and remained unchanged when rice was fully mature. The changes in 1000-grain weight of rice at different harvest times were consistent with those in the three regions of this study.

#### 3.3.2. Protein Content

**Figure 6:**
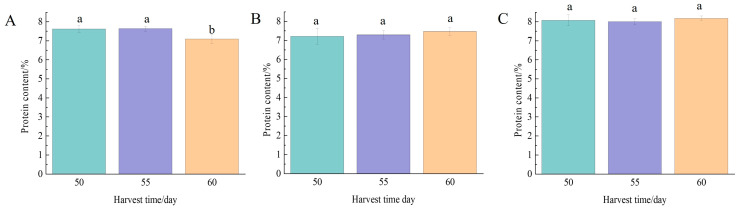
Protein content at different harvest times. (**A**) Nanjing, (**B**) Huai’an, (**C**) Lianyungang. Different lowercase letters indicate inter-treatment differences are significant at the 0.05 level.

Protein is an important component of rice and is closely related to the texture of rice. As illustrated in Figure 6, harvest time had a small effect on the protein content of rice. Nangeng 5718 rice planted in Nanjing had the highest protein content when harvested at 50, and 55 days after heading, which was significantly different from that harvested at 60 days after heading (*p* < 0.05). There was no significant difference in the protein content of Nangeng 5718 planted in Huai’an and Lianyungang at different harvest times (*p* > 0.05). Chen et al. [32] found that the protein content of rice reached the highest value at the end of ripening, and then showed a downward trend.

It was consistent with the protein content trend of Nangeng 5718 grown in Nanjing. It was also suggested that protein content was easily affected by factors such as light, temperature, and moisture. Moreover, high-temperature treatment at the fruiting stage increased the protein content of grains [33]. The accumulated temperature of the three harvest times in this area was generally higher at 1219–1409 °C, so the protein content reached the highest value 50 days after heading and then decreased. Fan et al. [34] found that the protein content of rice was mainly affected by genetic characteristics, and the delay of harvest had a certain effect on this index, but it was not significant. It was consistent with the changes in protein content of rice grown in Huai’an and Lianyungang in this study.

#### 3.3.3. Gelatinization Properties

**Figure 7:**
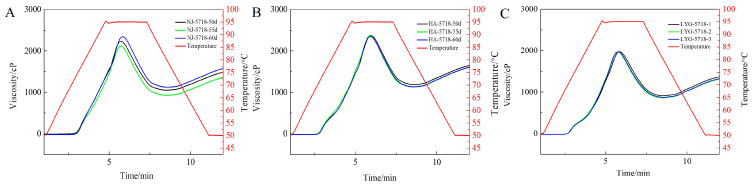
RVA curves at different harvest times. (**A**) Nanjing, (**B**) Huai’an, (**C**) Lianyungang.

The gelatinization properties of starch in rice are closely related to the cooking taste quality of rice. As can be seen from Figure 7 and Table 3, the RVA characteristic spectrum of Nangeng 5718 changed slightly with the change in harvest time; among them, the change range of Nangeng 5718 planted in Nanjing was larger, and the overall starch gelatinization characteristics of Nangeng 5718 rice planted in this area were greater than those planted in the other two regions.

The peak viscosity, minimum viscosity, final viscosity, and setback value were significantly increased compared with the previous two harvest times when Nangeng 5718 was harvested at 60 days after heading (*p* < 0.05). The results showed that rice had good water absorption and expansion during the cooking process, but it was more prone to aging, and the rice was harder after cooling compared with the previous two harvest times. There was no significant difference in other RVA characteristic values (*p* > 0.05). The accumulated temperature in Nanjing is high, and the change in RVA indicator is greater than Huai’an and Lianyungang. Nangeng 5718 planted in Huai’an had the highest setback value at 60 days, which was significantly different from that of rice at 50 days harvest (*p* < 0.05). Rice is easy to age. There was no significant difference in other RVA characteristic values (*p* > 0.05). Nangeng 5718 planted in Lianyungang had no significant difference in RVA characteristic values (*p* > 0.05). In this study, the change in harvest time had a great impact on setbacks. Song et al. [35] found that there were significant differences in the attenuation value and setback value of Yanfeng 47, Yangeng 228, and Yangeng 939 at different harvest times. Sui et al. [36] believed that rice with better taste quality usually had a larger attenuation value and a smaller setback value. Therefore, Nangeng 5718 in the three regions had low regeneration value and rice hardness and good gelatinization characteristics and rice cooking quality when rice were harvested at 50 days.

## 4. Conclusions

The accumulated temperature of different harvest times in Nanjing, Huai’an, and Lianyungang of Jiangsu Province changed significantly, and changing the harvest time of rice had an important impact on their qualities. There was no significant change in the 1000-grain weight of Nangeng 5718 at different harvest times. Harvest time had little effect on the browning of rice but had a more obvious effect on rice yield. Nangeng 5718 planted in Nanjing showed the highest rice yield at 50 days due to its suitable climate; however, rice planted in Huai’an reached its highest value at 55 days. Harvest time had little effect on the length-width ratio of brown rice, and the grain thickness increased with the increase in maturity. The immature grain rate decreased significantly with the further maturity of rice. Among them, the immature grain rate of Nangeng 5718 planted in Nanjing was 0–1.8%, and the accumulated temperature from heading to harvest in this area was 1219–1409 °C. Harvest time had a small effect on the protein content of rice. However, the accumulated temperature in Nanjing was relatively high. The protein content of rice in Nanjing reached the highest value at the end of ripening and then showed a downward trend. Harvest time had an effect on the gelatinization characteristics of Nangeng 5718, especially the difference in setback. The setback value of rice was the lowest at 50 days, and the gelatinization characteristics of rice were good. The RVA curve and RVA characteristic values of Nangeng 5718 planted in Nanjing were greater than those in Huai’an and Lianyungang due to the high accumulated temperature.

Harvested at 50 days after heading, the roughness rate, rice yield, 1000-grain weight, protein, and gelatinization characteristics were all at a good level. The overall qualities of the rice was better. Overall, harvest at 50 days after heading was the best harvest time to ensure the quality of rice. In this study, the optimal harvest time of Nangeng 5718 was determined through the change in rice quality. Future work will analyze the differences in rice at different harvest times from other perspectives to find the best harvest time.

## Figures and Tables

**Table 1:** Information about the Nangeng 5718 cultivar.

Planting Areas	Heading Time	Harvest Time	Heading to Harvest/Days	Accumulated Temperature/°C ^1^	Moisture/%
Nanjing	19 August 2023	8 October 2023	50	1219	9.45
	19 August 2023	13 October 2023	55	1314	10.29
	19 August 2023	18 October 2023	60	1409	9.05
Huai’an	22 August 2023	11 October 2023	50	1143	10.36
	22 August 2023	16 October 2023	55	1233	10.41
	22 August 2023	21 October 2023	60	1324	11.05
Lianyungang	3 September 2023	23 October 2023	50	1051	9.66
	3 September 2023	28 October 2023	55	1144	9.16
	3 September 2023	2 November 2023	60	1219	9.85

^1^ Accumulated temperature is the sum of the daily average temperature from heading to harvest. The data comes from the weather station.

**Table 2:** The length, width, and thickness of Nangeng 5718 at different harvest times.

Planting Areas	Harvest Time/Day	Length/mm	Width/mm	Thickness/mm	Ratio
Nanjing	50	5.3 ± 0.0 b	3.1 ± 0.1 a	2.2 ± 0.0 a	1.7 ± 0.1 a
	55	5.5 ± 0.0 a	3.1 ± 0.0 a	2.3 ± 0.0 a	1.8 ± 0.0 a
	60	5.4 ± 0.1 ab	3.1 ± 0.0 a	2.3 ± 0.0 a	1.8 ± 0.0 a
Huai’an	50	5.4 ± 0.0 a	3.2 ± 0.1 a	2.3 ± 0.0 b	1.7 ± 0.0 a
	55	5.5 ± 0.1 a	3.2 ± 0.0 a	2.3 ± 0.0 ab	1.7 ± 0.0 a
	60	5.4 ± 0.0 a	3.2 ± 0.1 a	2.3 ± 0.0 a	1.7 ± 0.0 a
Lianyungang	50	5.2 ± 0.0 a	3.2 ± 0.1 a	2.3 ± 0.0 b	1.7 ± 0.0 a
	55	5.3 ± 0.1 a	3.1 ± 0.0 a	2.3 ± 0.0 b	1.6 ± 0.0 b
	60	5.3 ± 0.1 a	3.2 ± 0.0 a	2.3 ± 0.0 a	1.7 ± 0.0 ab

Different lowercase letters after the same column of data indicate a significant difference between treatments at the 0.05 level.

**Table 3:** Gelatinization characteristics of Nangeng 5718 at different harvest times.

Planting Areas	Harvest Time/Day	Peak Viscosity/ cP	Trough Viscosity/cP	Breakdown/cP	Final Viscosity/cP	Setback/cP	Peak Time/min	Gelatinization Temperature/°C
Nanjing	50	2216.7 ± 25.5 b	1008.7 ± 35.6 b	1208.0 ± 46.8 a	1463.3 ± 35.9 b	454.7 ± 13.5 b	5.7 ± 0.0 a	68.4 ± 0.0 a
	55	2121.3 ± 29.3 b	928.7 ± 12.3 c	1192.7 ± 36.0 a	1403.7 ± 8.6 b	475.0 ± 10.4 ab	5.7 ± 0.1 a	68.4 ± 0.1 a
	60	2346.7 ± 72.7 a	1124.0 ± 22.5 a	1222.7 ± 50.2 a	1622.7 ± 49.3 a	498.7 ± 28.5 a	5.8 ± 0.1 a	68.3 ± 0.0 a
Huai’an	50	2377.3 ± 59.5 a	1188.3 ± 93.8 a	1189.0 ± 39.7 a	1689.3 ± 90.1 a	501.0 ± 3.0 b	6.0 ± 0.1 a	68.3 ± 0.1 a
	55	2382.3 ± 35.4 a	1133.7 ± 25.0 a	1248.7 ± 59.5 a	1644.3 ± 24.1 a	510.0 ± 9.7 ab	5.9 ± 0.1 a	68.4 ± 0.0 a
	60	2350.7 ± 82.8 a	1133.3 ± 73.0 a	1217.3 ± 24.8 a	1652.3 ± 78.2 a	519.0 ± 5.3 a	5.9 ± 0.0 a	68.4 ± 0.1 a
Lianyungang	50	1987.0 ± 64.1 a	999.3 ± 45.9 a	1076.3 ± 36.1 a	1406.7 ± 86.1 a	496.0 ± 10.4 a	5.8 ± 0.1 a	68.3 ± 0.0 a
	55	1999.0 ± 17.7 a	989.7 ± 43.5 a	1136.3 ± 65.0 a	1348.3 ± 80.3 a	485.7 ± 3.5 a	5.7 ± 0.2 a	68.4 ± 0.0 a
	60	1986.7 ± 69.7 a	919.7 ± 5.1 a	1113.0 ± 19.7 a	1361.3 ± 98.7 a	489.8 ± 10.6 a	5.7 ± 0.1 a	68.4 ± 0.0 a

Different lowercase letters after the same column of data indicate a significant difference between treatments at the 0.05 level.

## Data Availability

The original contributions presented in the study are included in the article, further inquiries can be directed to the corresponding author.
